# Supplementary figures and images for: Accurate and sensitive detection of microbial eukaryotes from whole metagenome shotgun sequencing
Source: Microbiome. 2021 Mar 3;9:58. doi: 10.1186/s40168-021-01015-y (PMC7931531; doi:10.1186/s40168-021-01015-y)

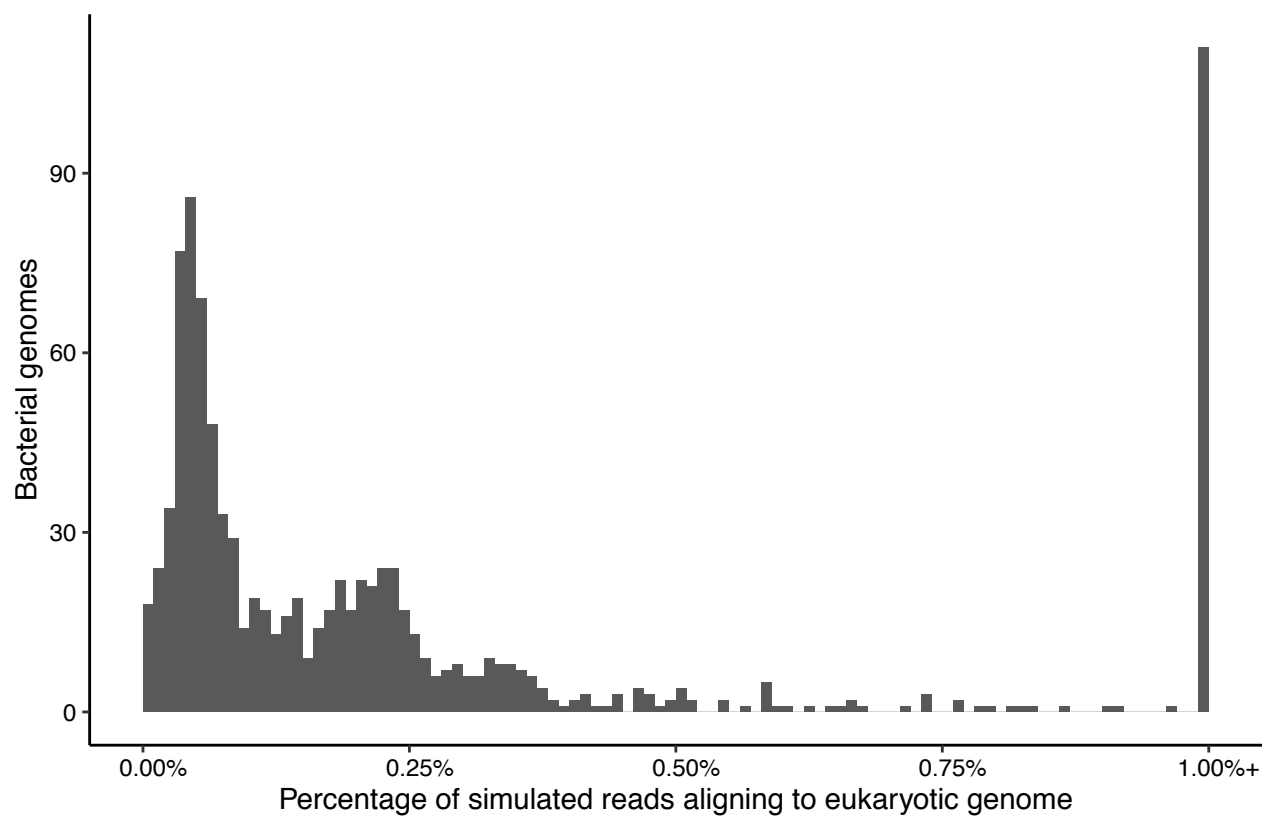

Supplement: Supplementary file 2 — Additional file 1: Figure S1. Percentage of simulated bacterial reads from 971 human gut microbiome bacteria that align to 2,449 eukaryotic genomes (see Figure 1a). [file 40168_2021_1015_MOESM2_ESM.pdf]

A

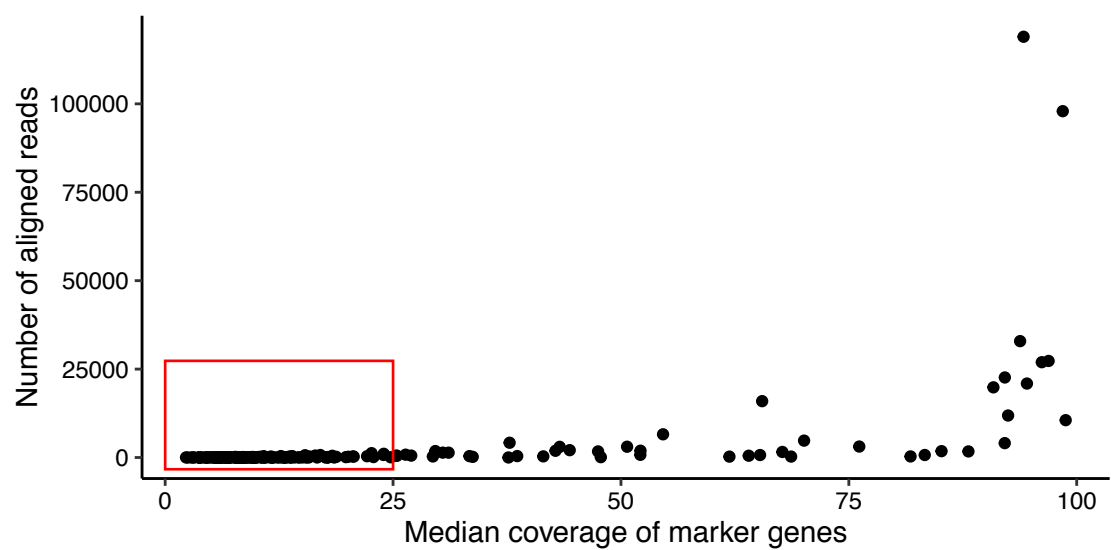

B

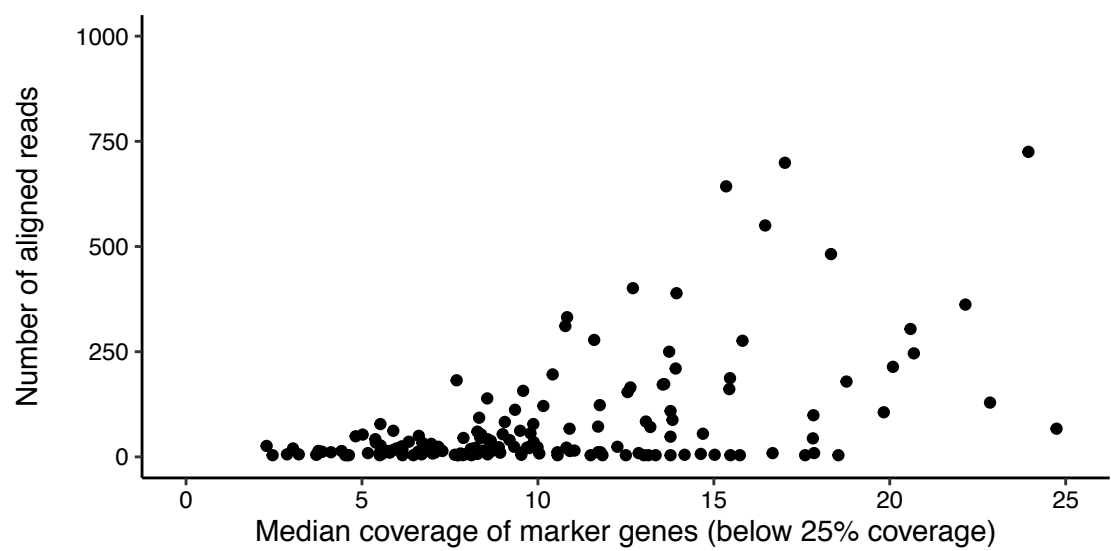

Supplement: Supplementary file 3 — Additional file 2: Figure S2. (A) Number of aligned reads to a species versus the median coverage of observed marker genes for that species. Median coverage and aligned read counts was calculated for each species within each of the 7 datasets analyzed in this work (see Methods). Red box indicates region depicted in (B). [file 40168_2021_1015_MOESM3_ESM.pdf]

Figure S1. Schematic of the EukDetect pipeline.

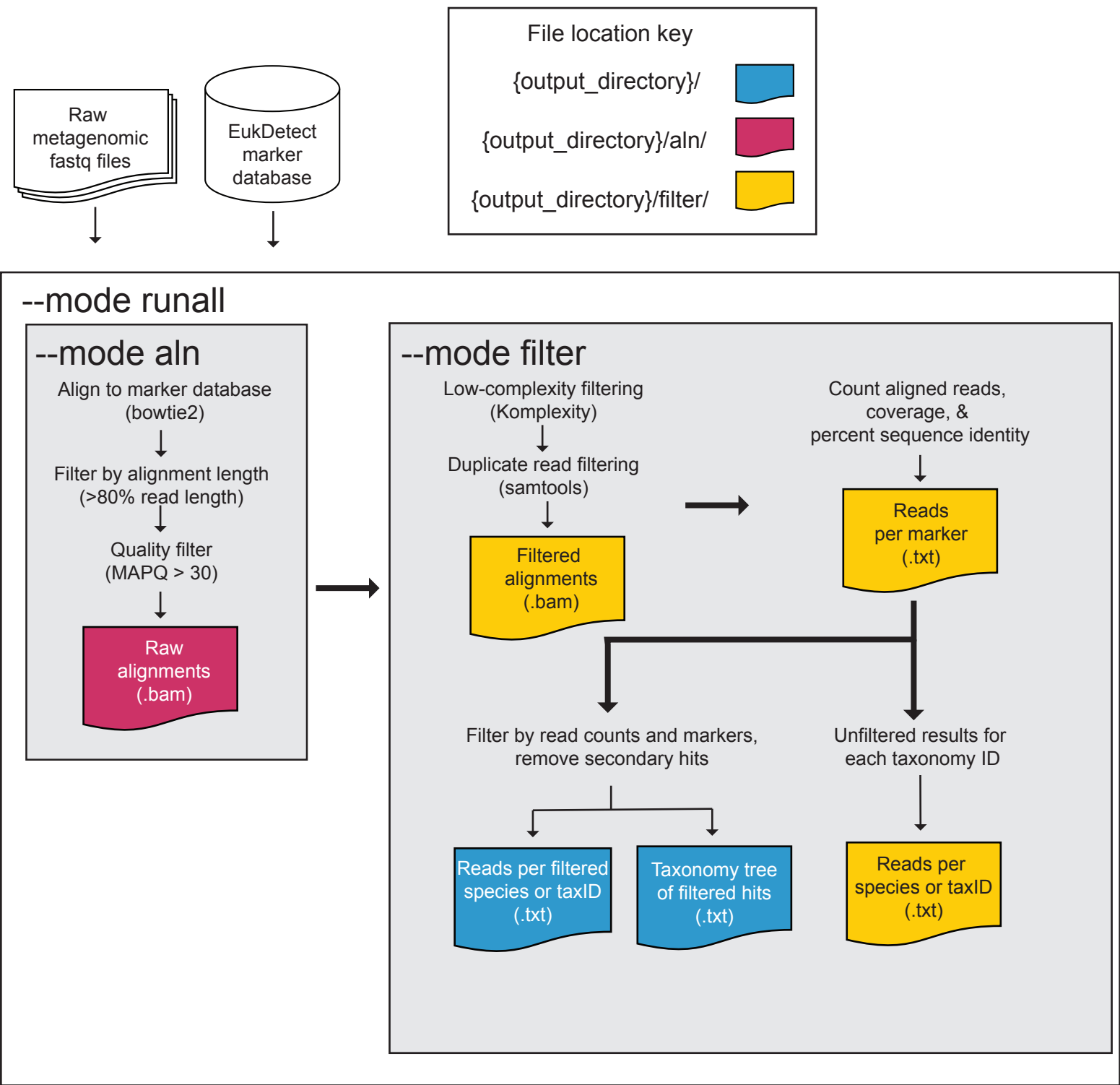

Supplement: Supplementary file 4 — Additional file 3: Figure S3. Schematic of the EukDetect pipeline. (PDF) [file 40168_2021_1015_MOESM4_ESM.pdf]
