# Supplementary material for: Accurate and sensitive detection of microbial eukaryotes from whole metagenome shotgun sequencing
Source: Microbiome. 2021 Mar 3;9:58. doi: 10.1186/s40168-021-01015-y (PMC7931531; doi:10.1186/s40168-021-01015-y)
Supplement: Supplementary file 5 — Additional file 4: Figure S4. Distribution of Blastocystis in the gastrointestinal tract taken from biopsies. Fungi were detected at all sites in the large intestine and terminal ileum, in both lumen and mucosal samples. Slashes indicate no Blastocystis detected in any samples from that site. (PDF) [file 40168_2021_1015_MOESM5_ESM.pdf]

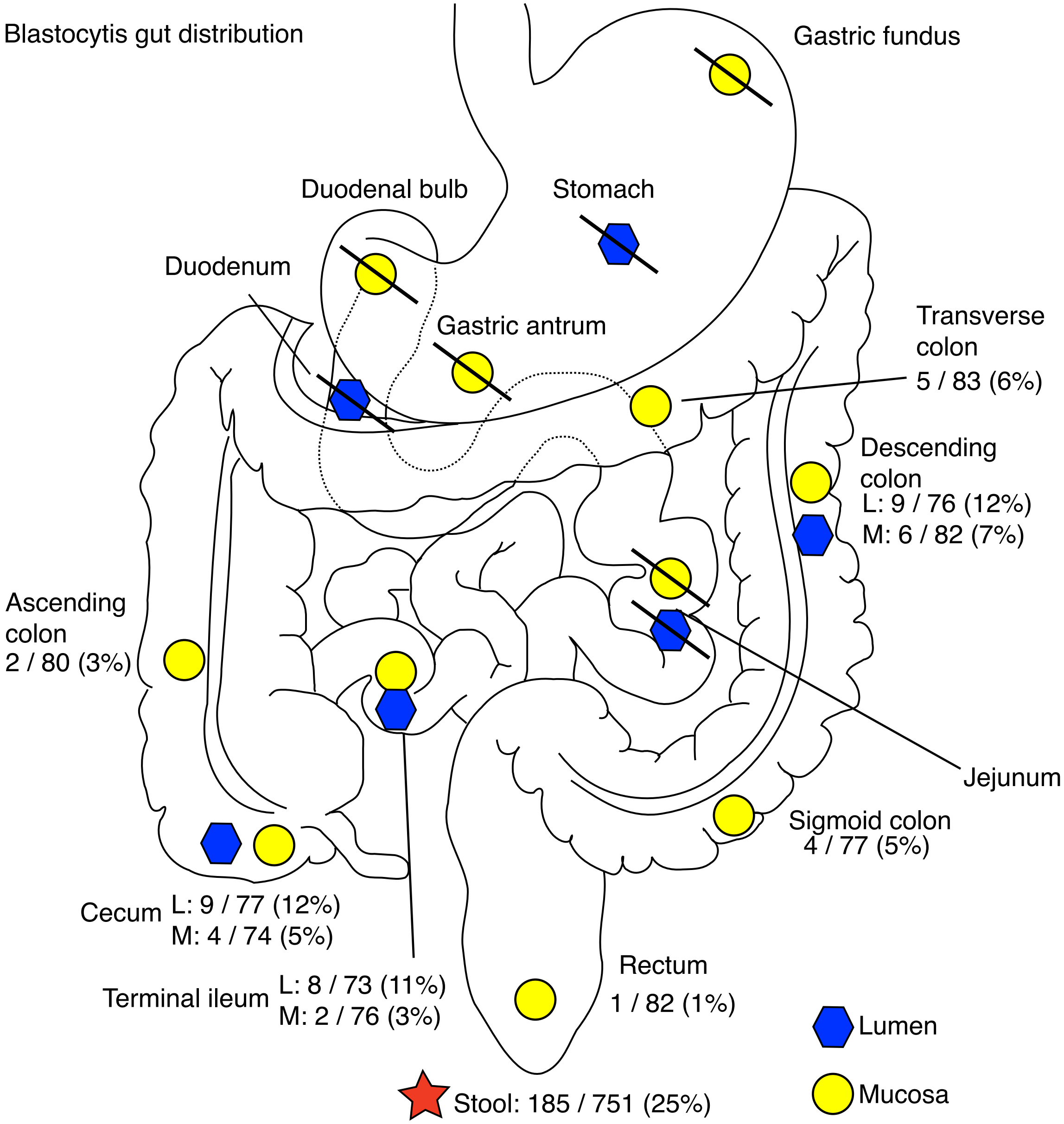

Figure S2. Distribution of *Blastocystis* in the gastrointestinal tract taken from biopsies. Fungi were detected at all sites in the large intestine and terminal ileum, in both lumen and mucosal samples. Slashes indicate no *Blastocystis* detected in any samples from that site.
