# Supplementary material for: Accurate and sensitive detection of microbial eukaryotes from whole metagenome shotgun sequencing
Source: Microbiome. 2021 Mar 3;9:58. doi: 10.1186/s40168-021-01015-y (PMC7931531; doi:10.1186/s40168-021-01015-y)
Supplement: Supplementary file 6 — Additional file 5: Figure S5. Distribution of fungal species in the gastrointestinal tract taken from biopsies. Fungi were detected at all sites in the large intestine in both lumen and mucosal samples, and in the lumen of the terminal ileum. One biopsy of gastric antrum mucosa in the stomach contained a Malassezia yeast. Slashes indicate no fungi detected in any samples from that site. (PDF) [file 40168_2021_1015_MOESM6_ESM.pdf]

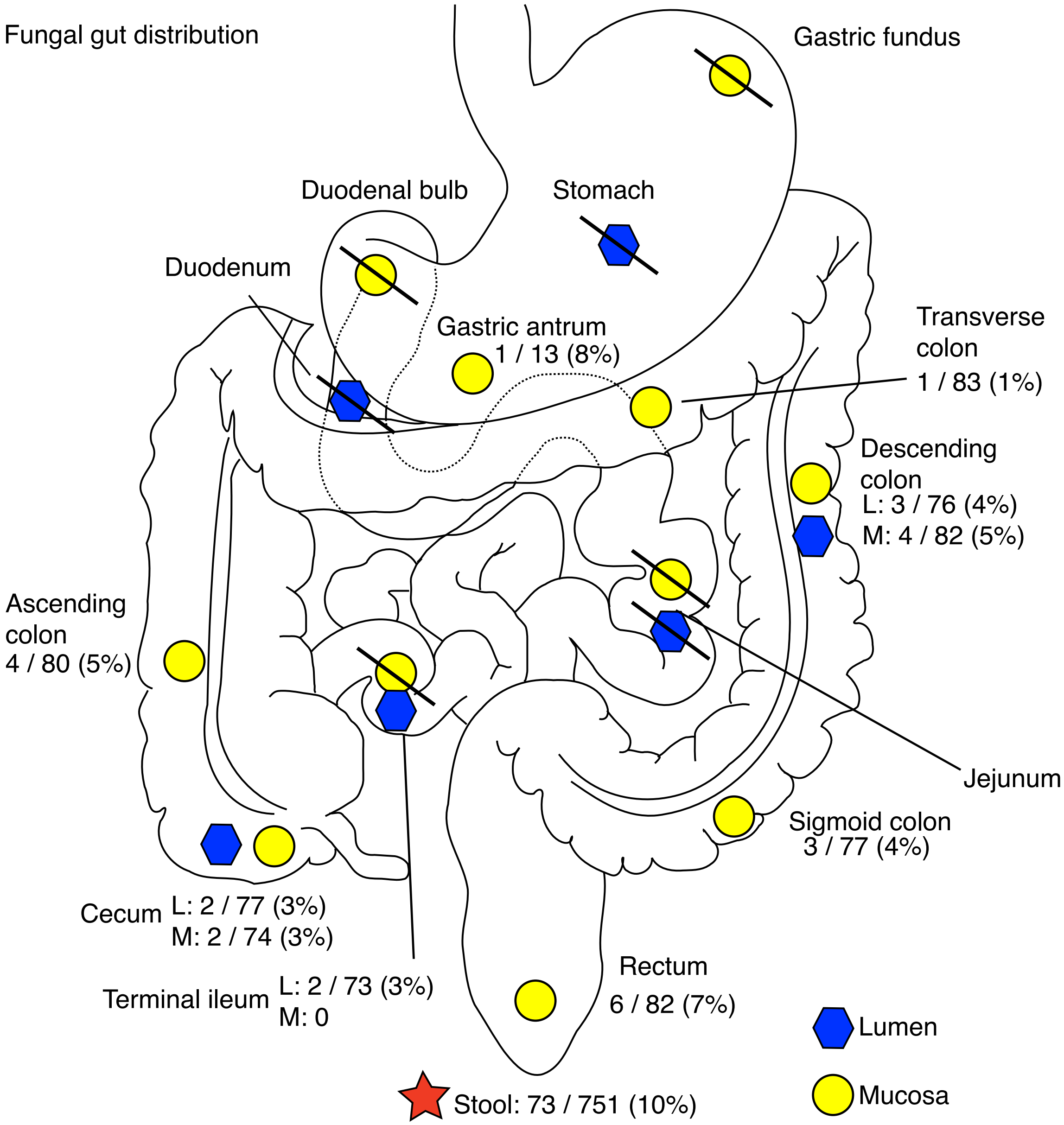

Figure S3. Distribution of fungal species in the gastrointestinal tract taken from biopsies. Fungi were detected at all sites in the large intestine in both lumen and mucosal samples, and in the lumen of the terminal ileum. One biopsy of gastric antrum mucosa in the stomach contained a *Malassezia* yeast. Slashes indicate no fungi detected in any samples from that site.
