# Supplementary material for: Accurate and sensitive detection of microbial eukaryotes from whole metagenome shotgun sequencing
Source: Microbiome. 2021 Mar 3;9:58. doi: 10.1186/s40168-021-01015-y (PMC7931531; doi:10.1186/s40168-021-01015-y)
Supplement: Supplementary file 7 — Additional file 6: Figure S6. (A) Counts of eukaryotic taxa observed in each Baltic Sea sample. (B) Counts of detected chloroplastid subgroups in different environments. (C) Counts of detected stramenopile subgroups in different environments. (D) Counts of detected metazoan subgroups in different environments. [file 40168_2021_1015_MOESM7_ESM.pdf]

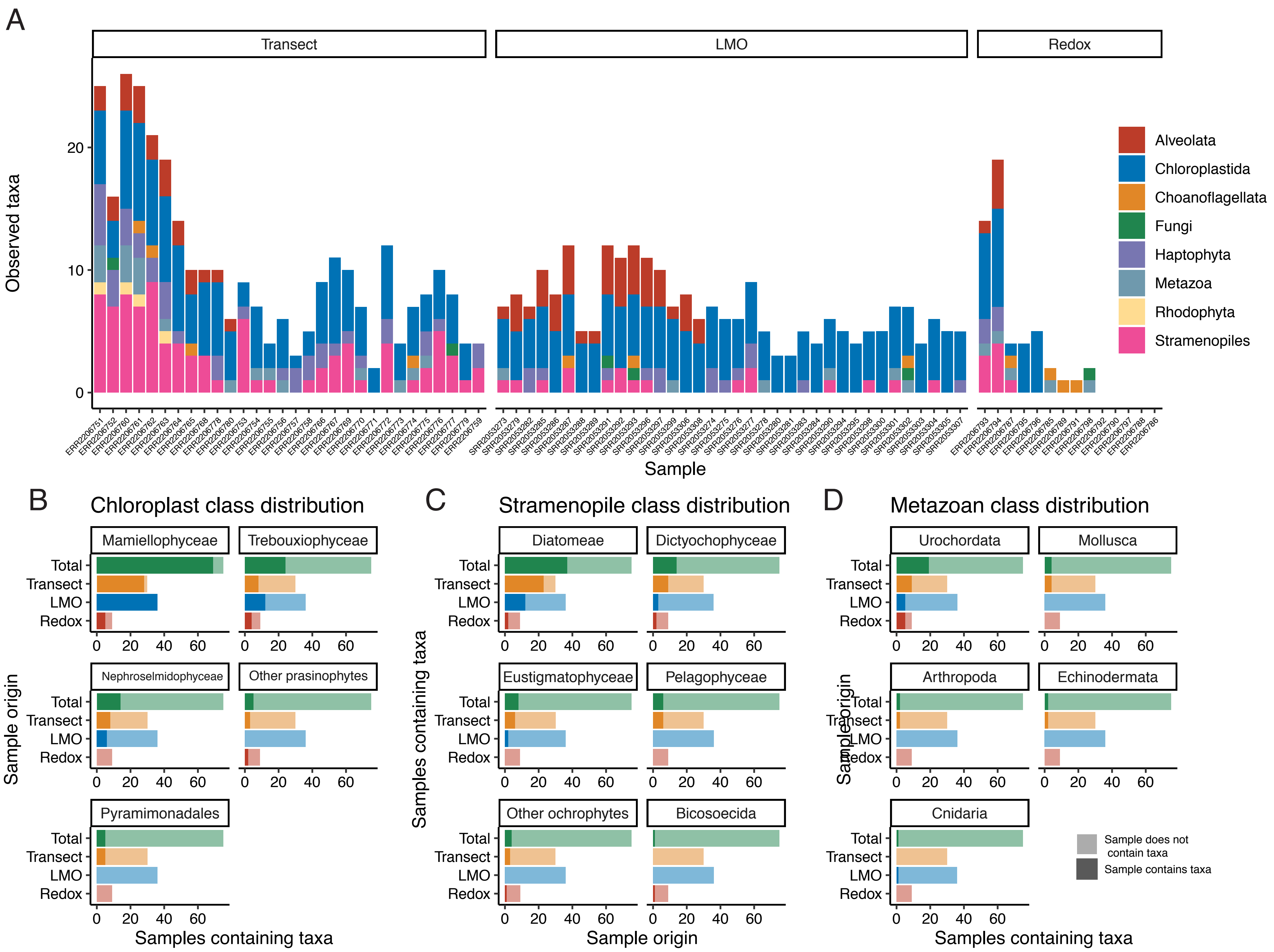

A
Transect
LMO
Redox
Observed taxa
0
10
20
Sample
Alveolata
Chloroplastida
Choanoflagellata
Fungi
Haptophyta
Metazoa
Rhodophyta
Stramenopiles
B
Chloroplast class distribution
Mamiellophyceae
Trebouxiophyceae
Nephroselmidophyceae
Other prasinophytes
Pyramimonadales
Sample origin
Samples containing taxa
C
Stramenopile class distribution
Diatomeae
Dictyochophyceae
Eustigmatophyceae
Pelagophyceae
Other ochrophytes
Bicosoecida
Samples containing taxa
Sample origin
D
Metazoan class distribution
Urochordata
Mollusca
Arthropoda
Echinodermata
Cnidaria
Sample origin
Samples containing taxa
Total
Transect
LMO
Redox
0
20
40
60
Sample does not contain taxa
Sample contains taxa
